# 

**Published:** 1898-10-08

**Authors:** 


					THE HOSPITAL. "The standard of highest purity."?Lancet. Oct. 8th, 1898.
CADBURY'S ^ WJT CADBURY'S ,
COOOA r\. Fm M OOCOA
ABSOLUTELY PURE. ? T NO DRUGS OR ALKALIES USED.
No. 628. Vol. XXV. [ftesgja Saturday,
Oct. 8th, 1898.
?-"'NUAMLKaub NORWICH H0SPIJA1
[SubB^ptioHTerms,] pRICE TWOPENCE.

				

